# Role and research progress of hematological markers in laryngeal squamous cell carcinoma

**DOI:** 10.1186/s13000-023-01335-7

**Published:** 2023-04-20

**Authors:** Hui Qi

**Affiliations:** 1 Nursing College, Shanxi Medical University, Taiyuan, 030001 Shanxi People’s Republic of China; 2 Shanxi Key Laboratory of Otorhinolaryngology Head and Neck Cancer, First Hospital of Shanxi Medical University, Taiyuan, 030001 Shanxi People’s Republic of China

**Keywords:** Hematological markers, Laryngeal squamous cell carcinoma, Inflammation, Tumor microenvironment, Prognosis

## Abstract

Laryngeal cancer is one of the most common malignant tumors of the head and neck, accounting for about 20%. Due to its high disability rate, the diagnosis and treatment of laryngeal cancer have always been the focus and difficulty of head and neck surgery. The outcome of cancer is affected not only by tumor-related factors but also by host-related factors, especially systemic inflammation, this is usually reflected by a variety of hematological markers. Studies have confirmed that there is a significant correlation between hematological markers and the occurrence, development, and prognosis of laryngeal squamous cell carcinoma (LSCC), and has a certain value in auxiliary diagnosis and prognosis prediction of LSCC. We reviewed various hematological markers related to LSCC aim to summarize the role and research progress of hematological markers in LSCC.

## Highlights

• The interaction between tumor and inflammation occurs through a variety of complex mechanisms, and inflammation plays an important role in every stage of carcinogenesis, including tumorigenesis, angiogenesis, inhibition of apoptosis, and tumor metastasis.

• More than 95% of metastatic malignant tumors have abnormal blood coagulation, and excessive blood coagulation caused by cancer may accelerate the progression and spread of the tumor.

• Platelet parameters play an important role in the occurrence and development of malignant tumors.

• Cancer-associated nutritional status plays a critical role in the progress of tumors and that these are also closely related to cancer-related cachexia.

• There is a significant correlation between hematological markers and the occurrence, development, and prognosis of laryngeal squamous cell carcinoma, but the research still has some limitations at present.

## Introduction

Head and neck tumors are the seventh-largest tumors in the world (5th in males and 12th in females) [1]. Laryngeal cancer is one of the most common malignant tumors of the head and neck, accounting for about 20% [2], squamous cell carcinoma is the most common. According to the global cancer statistics 2018 report [3], 177,000 cases of laryngeal cancer are diagnosed annually worldwide, and 95,000 deaths each year. The standard incidence of laryngeal cancer was 2.0/100,000, and the mortality was 1.0/100,000. As the early symptoms are not obvious, about 60% of the patients are already in the advanced stage when they seek treatment, missing the best time for treatment. Advanced patients have a high disability effect, which can cause dyspnea, dysphagia, and dysphagia, resulting in serious psychological problems and greatly affecting the quality of life [4, 5]. Although the overall incidence of laryngeal cancer has decreased over the past 40 years, the 5-year survival rate has decreased from 66 to 63%. Due to its high disability rate, the diagnosis and treatment of laryngeal cancer have always been the focus and difficulty of head and neck surgery.

In the past decade, the study of laryngeal squamous cell carcinoma (LSCC) has shifted from traditional clinicopathological factors to new biomarkers to more accurately describe the prognosis of the tumor and determine targeted therapeutic strategies [6–8]. At present, it is believed that the occurrence and development of LSCC is a combination of many carcinogenic factors, including long-term smoking or drinking, human papillomavirus (HPV) infection, air pollution such as polycyclic aromatic hydrocarbons, dust and mustard gas, occupational exposure such as asbestos, lack of nutritional factors, gastroesophageal reflux, disturbance of sex hormone metabolism and/or genetic susceptibility. More and more researchers begin to pay attention to potential biomarkers, such as genetic, immune, and hematological markers in peripheral venous blood [9–11]. Although serum tumor biomarkers cannot detect cancer at an early stage, they can be used to predict tumor metastasis, recurrence, and prognosis [12, 13]. Changes in tumor biomarkers levels usually occur within 2–3 months before imaging abnormalities can be detected.

At present, the TNM staging system established by AJCC and the International Alliance for Cancer Control is the most commonly used tumor staging standard in the world and the gold standard for prognosis evaluation and treatment of all kinds of malignant tumors [14, 15]. It plays a very important role in evaluating the therapeutic effect and prognosis of patients. However, heterogeneity is common among different patients with the same type of malignant tumor, which often makes it difficult to treat and evaluate the prognosis of malignant tumors. Since TNM staging can only be obtained after surgery, it is difficult to make a more personalized treatment plan for some patients to predict the prognosis before the operation [16]. The emergence of new biomarkers can help stratify high-risk patients and help make more accurate treatment plans [17]. Complete blood count (CBC) is a simple, convenient, and economical routine experiment to reflect systemic inflammatory response. The most important thing is that it can identify potential diseases with a high risk of disease recurrence and death, and can supplement TNM staging to stratify the survival of LSCC patients to identify high-risk patients. This article reviews the research progress of hematological markers in LSCC to provide ideas for further research.

## Hematological markers in LSCC

Several studies have assessed the role of hematological markers in the prediction of the progression and prognosis of LSCC, it can be roughly divided into the following four categories:

- Inflammatory markers: neutrophil/lymphocyte ratio (NLR), platelet/lymphocyte ratio (PLR), lymphocyte/monocyte ratio (LMR).
- Coagulation and fibrinolysis markers: prothrombin time (PT), activated partial thromboplastin time (APTT), fibrinogen (FIB), D-dimer (D-dimer).
- Platelet parameters: platelet count (PLT), platelet distribution width (PDW), mean platelet volume (MPV).
- Nutritional markers: albumin (ALB), globulin (GLB), C-reactive protein (CRP), hemoglobin (HB), albumin/globulin ratio (AGR), C-reactive protein/albumin ratio (CAR).

We will discuss the role and research progress of various hematological makers in LSCC.

## Role and research progress of hematological markers in LSCC

### Role and research progress of inflammatory markers in LSCC

In 1881, Professor Rudolf Virchow first noticed the white blood cells in tumor tissue and provided the first indication of a possible link between inflammation and cancer [18], and 15% to 20% of tumor deaths worldwide are related to inflammation [19]. At present, it is widely accepted that there is crosstalk between inflammatory response and cancer development. The interaction between tumor and inflammation occurs through a variety of complex mechanisms, and inflammation plays an important role in every stage of carcinogenesis, including tumorigenesis, angiogenesis, inhibition of apoptosis, and tumor metastasis [19–24]. Besides, growth factors and chemokines secreted by tumors regulate the inflammatory environment and cause a systemic inflammatory response. A large number of data show that the outcome of cancer is affected not only by tumor-related factors but also by host-related factors [25], especially systemic inflammatory response, which can be reflected by measuring hematological markers.

Current evidence suggests that within tumor tissue and outside cancer cells, host structures (such as extracellular matrix), non-immune cells (such as fibrous tissue cells), and immune cells, namely eosinophils, basophils, mast cells, lymphocytes, natural killer cells [26, 27], and dendritic cells, interact and contribute to a high immunosuppressive microenvironment. Besides, tissue hypoxia and necrosis can lead to complex interactions between tumor and non-specific host inflammation, which ultimately promote the progression of cancer. For the estimation of the systemic inflammatory response, neutrophils, lymphocytes, monocytes, and platelets have been used as prognostic factors for various malignant solid tumors [28]. Inflammatory prognostic scores calculated using immune cell-related values, such as NLR, PLR, and LMR have shown promise in LSCC [29–31]. (Table 1).

**Table 1:** Clinical studies in which predictive and prognostic roles of NLR, PLR, and LMR/MLR were evaluated in patients with LSCC

Year	Author	Case	Object	Inflammatory markers	Cut-off value	Conclusion
2014	Kum et al. [32]	209	BLL, PLL, LSCC	NLR	-	NLR could be a useful inflammatory marker to differentiate LSCC from BLL and PLL
2015	Tu et al. [33]	141	LSCC	NLR	NLR: 2.17	Elevated preoperative NLR was an independent predictor of poor prognosis for patients with LSCC after surgical resection
2016	Rachidi et al. [34]	543	HNSCC	NLR	-	NLR is a robust predictor of OS in oral, pharyngeal, and LSCC
2016	Wong et al. [35]	140	LSCC	NLR	-	Pre-treatment NLR may serve as a useful prognostic marker in LSCC
2016	Fu et al. [36]	420	LSCC undergoing total laryngectomy	NLR	NLR: 2.59	The NLR may be an independent prognostic marker for CSS and OS in patients with advanced LSCC undergoing total laryngectomy
2016	Zeng et al. [37]	125	patients with locoregionally advanced LC (cT3–4 N0–3 M0) treated with chemoradiotherapy	NLR	NLR: 3.0	Pre-treatment NLR is a useful prognostic marker in patients with locoregionally advanced LC treated with chemoradiotherapy
2016	Zhao et al. [38]	202	LC	NLR	NLR: 2.85	Preoperative NLR level influences the recurrence and cervical lymph node metastasis of LC and can be considered a prognosis factor of LC
2016	Wang et al. [39]	120	undergone post-operativeradiotherapy for LSCC	NLR, PLR	NLR: 2.79 PLR: 112	Markers of systemic and local inflammation, especially PLR was reliable prognostic factor in patients with LSCC
2017	Sumner et al. [40]	196	definitive treatment for cancers of the oropharynx or larynx	NLR	-	Higher pre-treatment NLR is prognostic of poor OS
2017	Kara et al. [31]	81	underwent surgery for LSCC	NLR, PLR	NLR: 2.04 PLR: 120.32	Pre-treatment high PLR is predictive factors of a survival rates in patients with LSCC. The high PLR increases mortality in patients with LSCC. A high NLR may predict local recurrence and decrease PFS in patients with LSCC
2017	Wu et al. [41]	405	laryngeal lesions diagnosed by pathology	NLR, LMR	-	LMR and NLR as a systemic inflammatory index have a certain reference value to differentiate LSCC from PLL and BLL
2017	Hsueh et al. [42]	979	LSCC	NLR, PLR, LMR	NLR: 2.40 PLR: 111.00 LMR: 3.50	Preoperative lymphocytes, NLR, PLR and LMR were significantly associated with cancer progression, DFS and CSS, and these hematological parameters could be considered independent prognostic values for patients with LSCC
2018	Du et al. [43]	654	LSCC	NLR	NLR: 3.18	Pre-treatment NLR was associated with the prognostic outcomes for patients with laryngeal cancer, and may assist to establish prognostic factors for these patients
2018	Cho et al. [44]	621	definitive radiotherapy for nasopharyngeal, oropharyngeal, hypopharyngeal, and LC	NLR	NLR: 2.7	Head and neck cancer tends to be more aggressive in patients with a high NLR, leading to a poorer outcome after radiotherapy
2018	Yilmaz et al. [45]	144	LC	NLR	-	NLR is a cheap and easily accessible marker which can be used as a prognostic faxtor in laryngeal cancer
2018	Zhong et al. [46]	413	T3-T4 LSCC	PLR	-	Change in PLR may serve as a useful prognostic predictor for patients with T3-T4 LSCC
2018	Mao et al. [47]	899	underwent laryngectomy for LSCC	PLR	PLR: 193.55	Patients with PLR > 193.55 experience poor outcomes and represent malnutrition, more advanced cancer stage
2018	Chen et al. [48]	361	LSCC	NLR, PLR, MLR	NLR: 2.45 PLR: 114 MLR: 0.21	The elevated preoperative NLR, PLR, MLR were significantly associated with worse survival and cancer progression. The preoperative NLR and postoperative MLR might be independent prognostic markers of OS and PFS in LSCC patients undergoing surgical resection
2019	Eskiizmir et al. [49]	229	Patients with benign, premalignant and malignant laryngeal neoplasms	NLR	NLR: 4	Pre-treatment NLR is a useful and reliable predictive and prognostic biomarker for patients with laryngeal carcinoma
2019	Gorphe et al. [50]	68	T3 LC treated with induction chemotherapy using a preservation protocol	NLR	NLR: 5	Patients treated with a preservation protocol, a high NLR, and anemia before induction chemotherapy were associated with shorter survival, independently of the response to chemotherapy
2019	Sheng et al. [51]	110	underwent surgical resection for LSCC	NLR	NLR: 2.22	Preoperative increased NLR was associated with reduced prognosis in patients with LSCC
2019	Marchi et al. [52]	113	LSCC, CG	NLR, PLR	-	NLR and PLR are significantly increased in LSCC
2019	kucuk et al. [53]	116	previously operated LSCC	NLR, PLR	NLR: 2.79 PLR: 112	No correlation was found between local and systemic inflammatory response
2020	Ye et al. [54]	197	oropharyngeal, hypopharyngeal and LC receiving multimodality treatment	NLR	NLR: 2.77	Pre-treatment NLR elevation is promising predictor of prognosis in patients with operable HNSCC
2020	Cai et al. [55]	203	underwent surgery for LSCC	NLR, PLR	NLR: 2.41 PLR: 110.94	NLR was valuable markers in predicting survival in patients with LSCC and may be used to inform clinicians in designing individual treatment strategies
2020	Chuang et al. [56]	141	hypopharyngeal cancer/LC	NLR, LMR	NLR: 2.95 LMR: 2.99	Pretreatment NLR is superior to LMR in predicting treatment response and clinical outcomes among patients with laryngeal/hypopharyngeal cancer treated by chemoradiation/radiation
2020	Xun et al. [57]	151	undergone surgery for LSCC	NLR, PLR, MLR	NLR: 2.2 PLR: 106 MLR: 0.18	Preoperative high NLR, PLR, and MLR were associated with poor prognosis
2021	Franz et al. [58]	60	treated with primary surgery for LSCC	NLR	NLR: 2.68	NLR was shown to be significant in predicting DFS and recurrence risk
2021	Zhou et al. [59]	180	male LHSCC	PLR	PLR: 112.5	Patients with LHSCC have abnormal high PLR, and a high pre-treatment PLR portends adverse survival
2021	Li et al. [60]	147	LSCC	NLR, PLR	NLR: 1.88 PLR: 117.36	Preoperative NLR, PLR are promising prognostic predictors for patients with LSCC
2021	Akkas et al. [61]	118	LC	NLR, PLR	NLR: 3.8 PLR: 158	High NLR, PRL have been identified as poor prognostic factors in DFS
2021	Sizer et al. [62]	452	BLL, PLL,MLL,CG	NLR, PLR	-	NLR, PLR were low in the CG and BLL groups and high in the MLL group
2022	Murad et al. [63]	168	LSCC	NLR	NLR: 2.02	Pretreatment NLR ≥ 2.02 was an independent prognostic factor for poor OS. BMI can change the prognostic capacity of NLR in patients with LSCC

#### Neutrophil/lymphocyte ratio (NLR)

NLR plays an important role in the occurrence, progression, and metastasis of the tumor microenvironment [32, 34, 37, 64–68]. Immune cell infiltration and immune response in the microenvironment are closely related to the tumors, while tumor-associated neutrophils in the microenvironment mainly promote tumor proliferation, metastasis, and tumor angiogenesis [21, 69]. Tumor-infiltrating lymphocytes such as CD8 + T cells can play an immune role in tumor patients, have a strong anti-tumor ability, and act as important anti-tumor immune cells [70]. Lymphocytopenia indicates a generalized state of immunodepression, and survival appears to be adversely influenced by depressed immune function. The number of CD4 + helper lymphocytes may decrease, and CD8 + suppressor lymphocytes may increase due to disturbed inflammatory response and may result in immunosuppression [71–73]. NLR can reflect the immune response of the body to the tumors. The increase of NLR in patients indicates the increase of inflammatory factors, the increase in the number of neutrophils and/or the decreasing number of lymphocytes may suppress lymphokine-activated killer cells [32, 74], and decrease the ability to kill tumor cells, and enhancement tumor invasion, resulting in poor tumor prognosis [34, 35, 75]. These may be the possible mechanisms for decreased survival in cancer patients.

Several studies on LSCC have reported that high NLR was associated with poor prognosis. The cutoff value for NLR varies from 1.88 to 5. Most of these studies have reported on the relationship between NLR and OS, DFS, PFS [33, 34, 40, 42, 48, 58, 61]. Kara et al. [31] analyzed high NLR may predict local recurrence and decrease PFS in patients with LSCC. While Kum et al. [32] reported that NLR could be a useful inflammatory marker to differentiate LSCC from the benign laryngeal lesion (BLL) and precancerous laryngeal lesion (PLL) patients. Marchi et al. [52] Reported that NLR is significantly increased in LSCC. Akkas et al. [61] and Xun et al. [57] demonstrated high NLR has been identified as a poor prognostic factor in DFS. Sizer et al. [62] reported that NLR was low in the control group (CG) and BLL groups, and high in the malignant laryngeal Lesion (MLL) group. Murad et al. reported that Pretreatment NLR ≥ 2.02 was an independent prognostic factor for poor OS and meanwhile they demonstrated the possible ability of BMI to change the prognostic capacity of NLR in patients with LSCC [63]. But kucuk et al. [53] Reported that no correlation was found between local and systemic inflammatory responses. Therefore, we suggested that LSCC patients be evaluated for NLR before and after treatment.

#### Platelet/lymphocyte ratio (PLR)

The increase of PLR value was first related to the increase of platelet ratio. A large amount of evidence shows that tumor cells can induce platelet activation, and in turn, activated platelets may also promote the growth of tumor cells [76], and the mechanisms are as follows: on the one hand, platelets can induce tumor spread and invasion by increasing angiogenesis, microvascular permeability and promoting tumor cell extravasation [77, 78]. On the other hand, the interaction between platelets and tumor cells can promote the proliferation of tumor cells and protect tumor cells from apoptosis [79]. From another point of view, high PLR levels in peripheral blood can also indicate a decrease in lymphocyte ratio. Lymphocytes and neutrophils account for the main part of total leukocytes and play an important role in systemic inflammation. They can inhibit or promote the progression of malignant tumors by regulating immune interactions in the microenvironment. As we all know, lymphocytes play a major role in the immune surveillance of malignant tumors, and they can inhibit the proliferation and metastasis of tumor cells [80]. The relative decrease of lymphocytes will make the body in an immunosuppressive state that inhibits the proliferation and metastatic activity of tumor cells, while the relative increase of neutrophils can provide the host with a microenvironment to promote tumor growth. The changes of both can indicate that the body has an insufficient immune response to the tumors.

Several studies have reported on the prognostic value of the preoperative assessment of the PLR. While a high PLR level has been reported associated with poor CSS and DFS [42, 61]. The cutoff value for PLR varies from 106 to 193.55. Kara et al. [31] reported that pre-treatment high PLR is a predictive factor of survival rates in patients with LSCC. The high PLR increases mortality in patients with LSCC. Wang et al. [39] reported that PLR was a reliable prognostic factor in patients with LSCC. Zhong et al. [46] reported that change in PLR may serve as a useful prognostic predictor for patients with T3-T4 LSCC. Mao et al. [47] reported that patients with PLR > 193.55 experience poor outcomes and represent malnutrition, more advanced cancer stage.

#### Lymphocyte/monocyte ratio (LMR)

The decrease of LMR reflects the imbalance between relatively low lymphocyte and high monocyte levels, which together form the tumor microenvironment. Tumor-infiltrating monocytes in the microenvironment promote tumor progression and metastasis mainly by promoting tumor cell formation, proliferation, and tumor angiogenesis, while tumor-infiltrating lymphocytes such as CD8 + T cells can play an immune role in tumor patients and have a strong anti-tumor ability [42, 81, 82]. However, tissue destruction and cell disintegration caused by tumor cell proliferation can cause a wide range of tumor-related immune responses in the body, which can be accompanied by a decrease in lymphocyte count [83]. The decrease of the number of lymphocytes leads to the weakening of immune response and the decrease of anti-tumor ability [84]. Some studies have shown that the increase of monocyte count is related to short survival time because monocytes can secrete a variety of inflammatory cellular molecules to promote tumor formation, angiogenesis, and distant metastasis [85].

Several studies have demonstrated that LMR/MLR was associated with a poor prognosis [57]. Hsueh et al. [42] reported that preoperative LMR was significantly associated with cancer progression, DFS, and CSS, and it could be considered independent prognostic values for patients with LSCC. Wu et al. [41] reported that LMR as a systemic inflammatory index has a certain reference value to differentiate LSCC from PLL and BLL. Chen et al. [48] reported that postoperative MLR might be independent prognostic markers of OS and PFS in LSCC patients undergoing surgical resection. Chuang et al. [56] reported that pretreatment NLR is superior to LMR in predicting treatment response and clinical outcomes among patients with laryngeal/hypopharyngeal cancer treated by chemoradiation/radiation.

### Role and research progress of coagulation and fibrinolysis markers in LSCC

The correlation between clotting factors and cancer can be traced back to more than a century ago. More than 95% of metastatic malignant tumors have abnormal blood coagulation, and excessive blood coagulation caused by cancer may accelerate the progression and spread of the tumor.

The main indexes reflecting blood coagulation and fibrinolysis in the clinic are PT, APTT, FIB, D-dimer, and so on. Some scholars think that the changes of PT and APTT are not lasting, so it is not suitable to be used as an index to observe clinical hypercoagulable states [86]. The contents of FIB and D-dimer in the blood are specific indicators reflecting the existence of hypercoagulable state and hyperfibrinolysis in the body. FIB is a protein synthesized by the liver. Its level is related to thrombin activity and is an important component involved in hemostasis and thrombosis. It is used as a scaffold for binding growth factor members, this combination can promote the proliferation of tumor cells and stimulate angiogenesis [87]. The increase of FIB can increase blood viscosity, promote thrombosis and cause thrombotic diseases [88]. Besides, FIB may promote inflammation by inducing excessive production of proinflammatory cytokines in malignant cells [89–91]. So elevated FIB is considered to be a manifestation of a prethrombotic state. At the same time, FIB is a stent for tumor angiogenesis, which contributes to the formation of tumor thrombus and distant metastasis of cancer cells [90–92].

D-dimer is a degradation product of FIB and a specific molecular marker of plasmin acting on cross-linked fibrin. When thrombin production increases and secondary hyperfibrinolysis occurred, the content of D-dimer increased, therefore D-dimer can be used as a molecular marker of hypercoagulable state and secondary hyperfibrinolysis, the increase of D-dimer reflects that the body is in a prethrombotic state or there is thrombosis in the body [93]. Because D-dimer is an ideal index to directly reflect the production of thrombin-bound plasmin, it can be used as an early sensitive index of coagulation dysfunction. In the tumor environment, tumor cells release various procoagulant substances, which directly or indirectly activate the coagulation cascade, resulting in thrombin production, fibrin formation, hypercoagulable state and thrombosis followed by hyperactivity of fibrinolytic system, which leads to the increase of D-dimer.

Few studies have shown that the relationship between LSCC and coagulation and fibrinolysis markers (Table 2). Sheng et al. [51] reported that FIB was a valuable marker in predicting survival in patients with LC and may be used to inform clinicians in designing individual treatment strategies. Cai et al. [55] reported that preoperative FIB was associated with reduced prognosis in patients with LSCC. However, considering the limited number of studies, the utility of coagulation and fibrinolysis markers remains to be established. Therefore, more studies are needed to further explore the relationship between LSCC and coagulation and fibrinolysis markers.

**Table 2:** Clinical studies in which predictive and prognostic role of FIB was evaluated in patients with LSCC

Year	Author	Case	Object	Coagulation and fibrinolysis markers	Cut-off value	Conclusion
2019	Sheng et al. [51]	110	underwent surgical resection for LSCC	FIB	FIB: 4 g/dL	Preoperative FIB was associated with reduced prognosis in patients with LSCC
2020	Cai et al. [55]	203	underwent surgery for LC	FIB	FIB: 3.05 g/L	FIB was a valuable marker in predicting survival in patients with LC and may be used to inform clinicians in designing individual treatment strategies

### Role and research progress of platelet parameters in LSCC

PLT plays an important role in the occurrence and development of malignant tumors [94]. It has been reported that 30% to 60% of malignant tumors are associated with an increase in PLT, even accompanied by thrombotic diseases, especially in the late stages of tumors [95, 96].

PLT can promote carcinogenesis in a variety of ways, such as providing mechanical protection to tumor cells during circulatory transport and promoting the transport and release of tumor molecules from their particles by enriching several biological activities in the tumor microenvironment [97]. Cytokines and growth factors contained in platelet alpha or dense granules can act as tumor-promoting signals and play a variety of roles in the tumor microenvironment, including promoting invasion and metastasis through active regulation of epithelium to mesenchyma [39, 98, 99], such as vascular endothelial growth factor (VEGF), epidermal growth factor (EGF), platelet-derived growth factor (PDGF), transforming growth factor β (TGF β), interleukin-1β (IL-1β), IL-8, and CXC-containing ligand 12 (CXCL12) [100–103].

Tumor cells can induce PLT aggregation and activate PLT, and activated PLT causes changes in platelet parameter PDW. Tumor cells can directly activate PLT through G protein-coupled receptors (GPCR) signal transduction, a process called tumor cell-induced platelet aggregation (TCIPA). Tumor cells release platelet mediators such as ADP, thrombin, thromboxane A_2_ (TXA_2_), and tumor-associated proteases to induce the activation and release of secondary mediators in activated platelets. ADP can bind to P2Y_1_ and P2Y_12_ receptors on the platelet surface and induce the activation and degranulation of platelet α-granules [104]. TXA_2_ activates platelets to convert arachidonic acid (AA) into prostaglandin E_2_ (PGE_2_) by binding to TP α or TP β receptors. PGE_2_ can further activate platelets by binding to EP receptors on the platelet surface [105]. Thrombin activates platelets through GPCR binding to protease-activated receptors (PAR) on the surface of platelets [106]. The other is signal transduction through the immunoreceptor tyrosine-based activation motif (ITAM). Platelet glycoprotein VI (GP VI) and C-type lectin-like 2 (CLEC-2) receptors are membrane glycoproteins expressed only in platelets and megakaryocytes, and GP VI is considered to be the main signal transduction receptor related to platelet activation on exposed collagen. The interaction between GP VI on platelets and collagen induces the activation and release of α-granules and dense granules [107]. Once platelets are activated, depressions will be formed on the surface to increase the activation area. The various units of actin are polymerized into various new shapes to form filamentous pseudopodia, flaky pseudopodia, balloon, or other shapes, depending on the external force on platelets, the extracellular signals recognized, and physiological signals [106]. The change of pseudopod size and shape will cause the change of PDW [108].

Mean platelet volume (MPV), the most commonly used measure of platelet size, is a surrogate marker of platelet activation [109]. MPV reflects the size of platelet volume, megakaryocyte proliferation, and platelet formation. Platelet distribution width (PDW) is a measure of platelet heterogeneity caused by the heterogeneous demarcation of megakaryocytes, reflects the uniformity of platelet volume and the distribution of platelet volume in the blood [92, 110, 111]. Recent reports demonstrated several cytokines, such as interleukin-6 (IL-6), granulocytes colony-stimulating factor (G-CSF), and macrophage colony-stimulating factor (M-CSF), regulate megakaryocytic maturation, platelet production, and platelet size [112, 113]. IL-6 promotes tumor angiogenesis, metastasis, and metabolism [114–118]. Furthermore, the cytokines G-CSF and M-CSF that be secreted by tumor cells could stimulate megakaryopoiesis and subsequent thrombopoiesis in cancer [119]. Another possible mechanism is that platelets promote the hypercoagulable state of cancer. Activated platelets create a procoagulant micro-environment that enables the tumor cells to cover themselves with platelets and evade the host immune system [120].

Several studies have reported that the role of platelet parameters in the progression and prognosis of LSCC (Table 3). Ye et al. [54] showed that pre-treatment PLT > 248 × 10^9^/L is a promising predictor of prognosis in patients with operable HNSCC. Pardo et al. [121] showed that PLT was significantly associated with survival in univariate analysis. However, in a multivariate analysis, it lost its prognostic capacity, limiting its utility as a prognostic marker in patients with HNSCC. Zhang et al. [112] reported that elevated PDW might be a novel prognostic marker in laryngeal cancer. Fu et al. [122] reported that the patients with laryngeal cancer have reduced MPV and increased PDW compared to the subjects without laryngeal cancer. In addition, MPV and PDW play different roles in laryngeal cancer from benign laryngeal disease. Guo et al. [123] reported that elevated PDW and decreased MPV could serve as independent biomarkers for worse survival in laryngeal cancer. But Sheng et al. [51] came to the opposite conclusion that preoperative increased MPV was associated with reduced prognosis in patients with LSCC. And Kara et al. [124] conclude that caution should be exercised when using these new hematological parameters, which can be affected by many factors. Therefore, more studies are needed to further explore the relationship between LSCC and platelet parameters.

**Table 3:** Clinical studies in which predictive and prognostic roles of PLT, MPV, and PDW were evaluated in patients with LSCC

Year	Author	Case	Object	Platelet parameters	Cut-off value	Conclusion
2017	Pardo et al. [121]	824	HNSCC	PLT	PLT: 250.05 × 10^9^/L	PLT was significantly associated with survival in univariate analysis. However, in a multivariate analysis it lost its prognostic capacity, limiting its utility as a prognostic marker in patients with HNSCC
2017	Zhang et al. [112]	241	LC	PDW	PDW: 16.7%	Elevated PDW might be a novel prognostic marker in laryngeal cancer
2018	Fu et al. [122]	618	LC, BLL, CG	MPV, PDW	-	The patients with LC have reduced MPV and increased PDW compared to the subjects without LC. In addition, MPV and PDW play different roles in LC from benign laryngeal disease
2019	Kara et al. [124]	453	BLL, PLL, and LSCC	PDW	-	Caution should be exercised when using these new hematological parameters, which can be affected by many factors
2019	Sheng et al. [51]	110	underwent surgical resection for LSCC	MPV	MPV: 9.5fL	Preoperative increased MPV was associated with reduced prognosis in patients with LSCC
2020	Ye et al. [54]	197	HNSCC	PLT	PLT: 248 × 10^9^/L	Pre-treatment PLT > 248 × 10^9^/L is a promising predictor of prognosis in patients with operable HNSCC
2021	Guo et al. [123]	640	LC	MPV, PDW	-	Elevated PDW and decreased MPV could serve as independent biomarkers for worse survival in LC

### Role and research progress of nutritional markers in LSCC

Emerging evidence suggests that cancer-associated nutritional status plays a critical role in the progress of tumors and that these are also closely related to cancer-related cachexia (Table 4) [125–127].

**Table 4:** Clinical studies in which predictive and prognostic roles of HB, CRP, CAR, and AGR were evaluated in patients with LSCC

Year	Author	Case	Object	Nutritional markers	Cut-off value	Conclusion
2001	Nguyen-Tan et al. [128]	223	T3-4 LSCC	HB	HB: 12.5 g/dL	HB levels > or = 12.5 g/dL during radiotherapy was a favorable prognostic factor for OS
2004	Haugen et al. [129]	214	stage I-IV LC	HB	HB: 137.5 g/L	Preradiotherapy HB level, predicts locoregional control and survival in patients with LC treated with radiotherapy
2012	Zeng et al. [130]	57	locoregionally advanced LC treated with chemoradiotherapy	CRP	CRP: 8 mg/L	The elevation of CRP before treatment predicts a poor prognosis in patients with locoregionally advanced laryngeal carcinoma treated with chemoradiotherapy
2017	Chen et al. [131]	241	LSCC	AGR	AGR: 1.28	Low preoperative AGR could serve as a valuable and easily assessed blood-based indicator to predict the prognosis of LSCC patients
2017	Yu et al. [132]	129	LSCC	CAR	CAR: 0.047	Pretreatment CAR may be a significant prognostic marker in LSCC
2019	Zhou et al. [133]	232	LSCC	AGR	AGR: 1.31	AGR might be a promising marker to better predicting prognosis of LSCC patients
2019	Kuboki et al. [134]	56	Underwent total laryngectomy or total pharyngolaryngectomy	CAR	CAR: 0.32	CAR may be a novel and useful indicator for predicting postoperative outcomes in patients with hypopharyngeal and laryngeal cancer
2019	Fu et al. [135]	61	LC patients received radiotherapy	CRP	CRP: 10 mg/L	CRP may be used as a prognostic indicator for laryngeal cancer patients treated with radiotherapy
2020	Gorphe et al. [50]	68	T3 LC treated with induction chemotherapy using a preservation protocol	HB	-	In LC, patients treated with a preservation protocol, anemia before induction chemotherapy was associated with shorter survival, independently of the response to chemotherapy
2020	Sahin et al. [136]	432	Underwent laryngeal microsurgery because of benign and premalignant lesions or malignancy	CRP	-	CRP was shown to be increased in patients with laryngeal malignancies
2021	Tanoue et al. [125]	46	R/M HNSCC treated with nivolumab	CAR	CAR: 0.30	pretreatment CAR was an independent marker of survival and efficacy of nivolumab in recurrent or metastatic HNSCC patients, and that the CAR was a better predictor than the NLR

ALB and GLB are two important components of systemic inflammation, and the combination of these two markers (AGR) has been reported to be significant and validated in several types of cancers [137–140]. Studies have shown that AGR is closely related to the occurrence and progression of LSCC. ALB is the most abundant plasma protein, accounting for about 50% of the total protein content [141]. As a chronic phase protein, serum ALB is not only the most direct laboratory index to evaluate the nutritional status of protein, but also a sign of systemic inflammatory response. It can promote the release of IL-1, IL-6, TNF-α, and acute reactant, help stabilize DNA replication and cell growth, regulate body response, enhance natural immunity, and prevent malignant diseases [142]. Inflammatory response and tumor status can affect the concentration of serum ALB, low ALB levels may weaken the immune system, increase the chances of infection, and further accelerate the development of malignant tumors [143–145]. Serum ALB has been shown to have protective effects, such as maintaining physiological homeostasis, antioxidant activity, anti-inflammatory effects, and preventing apoptosis [145]. The study found that the level of ALB can reflect the nutritional status of cancer patients. In the case of low ALB, the cellular immunity and phagocytic function of patients are low, and the immune system is relatively fragile, easy to infect, affecting the effect of treatment [146]. GLB is composed of all pro-inflammatory proteins in plasma, including acute phase proteins and immunoglobulins. The accumulation of pro-inflammatory proteins leads to the increase of GLB levels, reflecting the inflammatory response of the body and continuous exposure to different pro-inflammatory factors, it can also promote tumor growth and proliferation. The decrease of AGR indicates that the level of serum ALB is low, the level of GLB is high, and the overall state of the body is poor [133]. Several studies have reported on the association of low AGR with poor prognosis in patients with LSCC. Zhou et al. [133] reported that AGR might be a promising marker for better predicting the prognosis of LSCC. Chen et al. [131] reported that Low preoperative AGR could serve as a valuable and easily assessed blood-based indicator to predict the prognosis of LSCC. However, considering the limited number of studies, the utility of AGR remains to be established.

CRP is a non-specific phase protein in the acute phase that is mainly synthesized and secreted by the liver. When there is acute rejection, bacterial infection, and operation, the synthesis of CRP in hepatocytes is significantly increased, it can activate complement, participate in apoptosis, promote granulocyte and macrophage phagocytosis, and can predict or diagnose malignant tumors [147, 148]. At the same time, as the most sensitive biomarker of inflammation, the increase of CRP level is one of the reasons for the poor prognosis of patients with malignant tumors [130, 149]. Several studies have shown that the relationship between LSCC and CRP. The cutoff value for CRP varies from 8 mg/L to 10 mg/L. Zeng et al. [130] and Fu et al. [135] reported that CRP may be used as a prognostic indicator for laryngeal cancer patients treated with radiotherapy or chemoradiotherapy. Sahin et al. [136] reported that CRP was shown to be increased in patients with laryngeal malignancies. However, considering the limited number of studies, the utility of CRP remains to be established.

Studies have shown that cancer patients may develop anemia. Growing tumors induce thrombocytosis by secreting inflammatory cytokines, which may also lead to myelosuppression, and abnormal iron metabolism, leading to tumor-induced anemia. Lower hemoglobin (HB) levels can lead to tumor hypoxia, which leads to faster tumor growth. In addition, anemia can also promote angiogenesis and genomic mutations in cells. Several studies have shown that preoperative hemoglobin levels are related to the prognosis of tumors [150]. Tumors can affect hematopoiesis by infiltrating into the bone marrow through massive tumor bleeding or by producing pro-inflammatory cytokines and free radicals that damage hematopoietic progenitor cells. Anemia may also be associated with paracrine signal transducers that affect the production of red blood cells, such as pro-inflammatory cytokines IL-1, and TNF-α. Several studies have reported on the relationship between HB level and the prognosis of LSCC. The cutoff value for HB varies from 125 g/L to 137.5 g/L. These studies reported that Hb level had a significant impact on prognosis in patients with LC treated with radiotherapy and chemotherapy [50, 128, 129].

It is stated that the C-reactive protein/albumin ratio (CAR) is a new independent prognostic factor for total survival and disease-free survival in laryngeal cancer [132]. IL-6 signals inhibit several immunocompetent cell activation in the tumor microenvironment [151]. It also induces LSCC cells to invade and metastasize and has been associated with recurrence and survival in LSCC. Locally or systemically elevated IL-6 levels have been reported to be associated with increased CRP concentrations in various cancers. Furthermore, IL-6 also induces cachexia by altering the metabolism of lipids and proteins [125, 152]. Few studies have reported on the relationship between CAR and the prognosis of LSCC. The cutoff value for CAR varies from 0.047 to 0.32. Tanoue et al. [125] reported that CAR was a better predictor than the NLR in HNSCC. Yu et al. [132] and Kuboki et al. [134] demonstrated that CAR may be a significant prognostic marker in LSCC. However, these results need to be validated in further studies.

## Conclusions and perspectives

Cancer affects various parts of the body through the systemic immune response, including changes in hormones, the number and ratio of white blood cells and platelets, and C-reactive protein or albumin levels under the influence of neuroendocrine metabolism, hematopoietic function, and protein and energy metabolism, respectively. Although oncology has made great progress, there is still a lack of effective markers to predict the effect of treatment, so there is an urgent need for non-invasive detection based on body fluid samples (such as blood, urine, and saliva) for rapid diagnosis or treatment monitoring. The peripheral blood test has the advantages of simplicity, convenience, economy, and repeatability. Therefore, a comprehensive understanding of hematological markers is helpful for tumor diagnosis, the guidance of targeted treatment, and the monitoring of therapeutic efficacy and drug resistance.

In this review, we discussed the applicability of NLR, PLR, LMR, FIB, PLT, PDW, MPV, CRP, HB, AGR, and CAR in patients with LSCC. These hematological markers have proven to be useful during preoperative evaluations in the treatment and risk assessment of patients with LSCC. But the research still has some limitations at present. First of all, most of the studies are retrospective, single-center studies, with a small sample size, which may introduce biases related to retrospective studies. Although many studies have recorded detailed data and follow-up results, prospective studies will help to better evaluate the prognostic factors of patients with laryngeal cancer. Therefore, the conclusions still need to be verified in prospective studies with larger sample sizes. Secondly, many other factors affect hematological markers, such as acute undetected infections and hematological diseases, which affect the accuracy of prognosis prediction based on hematological markers. Third, there is a lack of a clear method to obtain the best cut-off value of hematological markers, and the current recommended critical value is empirical and is determined by the simplicity of the calculation and the relatively good balance of the number of patients in the upper and lower groups. So there is a need to develop uniform classification criteria with a consensus regarding the use of these hematological markers in clinical settings. We believe that large prospective studies are needed on this subject based on observations of this retrospective review to further explore the relationship between hematological markers and laryngeal squamous cell carcinoma.

## Data Availability

All materials, data, code, and associated protocols will be promptly available to readers without qualifcations or restrictions. The datasets used and/or analyzed during the current study are available from the corresponding author on reasonable request.

## Abbreviations

HNSCC: Head and neck squamous cell carcinoma
LHSCC: Laryngeal/hypopharyngeal squamous cell carcinoma
LSCC: Laryngeal squamous cell carcinoma
LC: Laryngeal carcinoma
HPV: Papillomavirus
CBC: Complete blood count
NLR: Neutrophil/lymphocyte ratio
PLR: Platelet/lymphocyte ratio
LMR: Lymphocyte/monocyte ratio
PT: Prothrombin time
APTT: Activated partial thromboplastin time
FIB: Fibrinogen
PLT: Platelet
PDW: Platelet distribution width
MPV: Mean platelet volume
ALB: Albumin
GLB: Globulin
CRP: C-reactive protein
HB: Hemoglobin
AGR: Albumin/globulin ratio
CAR: C-reactive protein/albumin ratio
OS: Overall survival
DFS: Disease-free survival
PFS: Progression-free survival
CSS: Cancer-specific survival
BLL: Benign laryngeal lesion
PLL: Precancerous laryngeal lesion
MLL: Malignant laryngeal Lesion
CG: Control group
